# Acute Inflammation of Psoas Magnus Muscle

**Published:** 1867-12

**Authors:** J. W. Grosvenor

**Affiliations:** Providence, R. I.


BUFFALO

### Medical and Surgical Journal.

VOL. VII. DECEMBER, 1867. No. 5.

### Original Communications.

ART. I.—*Acute Inflammation of Psoas Magnus Muscle.* BY J. W. GROSVENOR, M. D., *Providence, R. I.*

Acute inflammation of Psoas Magnus Muscle is a very rare disease. In searching medical literature I find only a single case reported in detail, and that by Dr. C. W. Parsons of this city, in the *Boston Medical and Surgical Journal* of September 10th, 1851. A case is mentioned by Prof. Gross in the 1866 edition of his surgery. On page 594 he says: "An instance has been reported of a young man who died from rupture of the psoas muscle, death having been preceded by severe inflammation and infiltration of pus." In a letter to Dr. Anthony of this city, Prof. Gross regrets his inability to refer to the particular case.

Prof. Willard Parker informs me by letter that he has the notes of two unpublished cases which have occurred in his practice. I hope we may soon see them published.

The following case has lately come under my observation:

Lemuel Grosvenor Perry, a student, 19 years of age, strong and healthy from birth, while playing ball on June 27th, 1867, felt something "give way" in his right side. Soon after returning home, on the same day, he was taken sick with vomiting and quite a sharp pain in the right lumbar region. Tongue was slightly furred, bowels constipated, pulse full, and about 90 per minute.

A cathartic of magnesia in combination with charcoal, acted promptly, by which the pain was considerably relieved, and the vomiting entirely. On the morning of June 29th, patient had quite a severe chill. For the four or five days following there was but little change in the symptoms—pulse fair and about 80 per minute; tongue slightly brown and moist; stomach uncomfortable, tendency to diarrhœa; the pain in the right lumbar region continuing, though not severe. July 4th, a swelling and hardness were observed over the seat of the pain. It was circumscribed and covered a space of about four square inches, its centre being on a vertical level with the anterior superior spinous process of the ilium. For two or three days it became a little more prominent and then remained in *statu quo*. Patient found urination difficult without standing. He could not fully extend his right leg, kept the right thigh flexed on the abdomen and rotated outwards, moved from side to side in bed with difficulty and pain; when on his feet assumed a stooping posture, with the body inclined towards the right side. Abdomen not swollen and not tender on pressure, except in right lumbar and right iliac regions. From the 4th to the 12th, patient was comfortably sick, was able to get up without assistance every day; his pulse fair, the general symptoms being the same as during the few days previous to the appearance of the swelling. At 3 A. M. of the 12th, he was seized with a severe pain and excessive vomiting of a greenish-looking material; cold, clammy perspiration followed; pulse became very rapid and feeble Vomiting continued, with slight abatement, till death at 6½ o'clock P. M. of the same day.

Treatment previous to the sinking stage on July 12th, consisted in a cathartic at the outset of the disease, as already mentioned, injections of starch and laudanum to control the diarrhœa, anodynes sufficient to relieve pain and procure sleep at night, animal broths and some fruits, occasionally brandy and water. The tumor was painted with tinct. iodine for several days, and leeches applied to it on the 11th. In the stage of collapse treatment consisted in morphia injected hypodermically and iced champagne, to which was added a few drops of chloroform.

During his sickness the patient, who was under the medical care of his father, Dr. Perry, was seen by Drs. Peckham and Parsons of this city, and Dr. Clapp of Pawtucket.

Autopsy by Dr. Mason, 72 hours after death. Greater omentum slightly adherent to small intestines, which were considerably congested. Lower part of ascending colon and appendix vermiformis adherent to abdominal wall. A few fibres of psoas magnus muscle were rough and broken down, and the muscle itself dissected up along its posterior surface and the peritoneum separated from its anterior surface. The anterior crural nerve running along the outer border of the muscle was separated from all attachments for a distance of six inches. About three pints of purulent fluid in peritoneal cavity. The rupture of the peritoneum was apparently between the stomach and liver. Right kidney healthy. No diseased bone discovered. Undoubtedly inflammation commenced at that part of the psoas magnus muscle where the fibres were broken down, an abscess followed, and pus as it was formed burrowed under the muscle, dissected up the peritoneum, and finally burst into the peritoneal cavity on the morning of July 12th, at the time when the alarming change in the symptoms occurred.

I have seen no account of this disease in the English language. In Copland's Medical Dictionary is an article on "Inflammation and Suppuration of the Psoas Muscles." The author mentions psoitis or one of its synonyms, but he evidently refers to psoas abscess, which is a *chronic*, not an *acute* disease. The only article on this subject which I have seen is in the Dictionnaire de Medicine, vol. xxvi, under the heading "Psoite." The rarity of the disease and the meager amount of literature upon it may justify me in presenting a *resumé* of this article.

*Causes of Psoitis.*—Falls, blows on the lumbar region and pelvis, violent motion of body backwards and forwards on lower extremities, raising heavy weights, very severe exercise. Ferrus, the author of the article, thinks rheumatism has a great influence on the development of the disease.

*Symptoms.*—Pain in lumbar region which soon extends to groin and thigh—usually intolerable, rarely almost nothing. Extension and flexion of the thigh greatly increases the pain. Walking difficult or impossible. If patient walks trunk is strongly inclined forwards. Engorgemeut of inguinal glands. As disease progresses pain becomes severer, and lower extremity of affected side is constantly flexed and slightly turned outwards. An attempt at extension or rotation gives excessive pain. This position of the limb is ordinarily the most characteristic sign of psoitis. Sometimes there is numbness in the limb. Fever declares itself, digestive organs become deranged; sometimes nausea and vomiting, often diarrhœa, rarely constipation; urine is sometimes purulent, sometimes colored with blood. At last in the groin or lumbar region appears a tumor more or less extensive, fluctuating, not changing color of skin—not painful on pressure, drawing back into interior of abdomen. In this tumor whether it opens spontaneously or is opened by an instrument, is found more or less pus. Hectic fever follows, pulse becomes small and frequent, a cough appears, colliquative diarrhœa supervenes and the patient dies of marasmus.

*Pathological Anatomy.*—The muscle is found in three conditions. First, the muscle is entirely preserved, but softened; in color like lees of wine, infiltrated with black blood, and easily torn. Secondly, vestiges of the muscle remain of the consistency of pulp, blackish in color, and of a disagreeable odor. Thirdly, the muscle is completely destroyed by suppuration. The secretion is not pure pus, but a mixture of pus and muscular fibres, not entirely broken down. Usually suppuration extends towards the surface, but Ettmuller and Withmore have each reported a case in which the purulent matter found its way into the intestines and appeared in the evacuations. These two patients died, and the autopsies revealed a communication between the disease and colon in both cases. Sometimes the secretion follows the psoas and iliacus muscle as far as their insertion into the lesser trochanter, and infiltrates the muscles surrounding the coxo-femoral articulation.

*Diagnosis.*—The symptom which may be considered pathological, is flexion of the lower extremity upon the trunk in the direction of the fibres of the psoas muscle. This sign, taken in connection with the acute pain caused by rotating the limb will enable us to make out a diagnosis in a large majority of cases. Sometimes a diagnosis is very difficult. From abscess due to change in lumbar vertebræ, it may be distinguished by the rapidity of its course, by absence of deformity in vertebral column, by the impossibility of extending the leg, and of easily executing the movement of rotation. Nephritis, although resembling psoitis in the seat of the pain, differs from it in not preventing the movements of extension and rotation of the limb. In hernia the absence of fluctuation in the tumor, digestive troubles, absence of lumbar pain exclude the existence of psoitis. Sometimes there is great difficulty in diagnosticating between coxalgia and psoitis. In the latter as in the former the pain extends along the thigh, and sometimes as far as the knee, and also there is sometimes the same difficulty of rotation in one as in the other. Still the principal seat of the pain at the outset of the disease being in the lumbar region in psoitis and coxalgia in the external iliac fossa will usually prevent any mistakes. (From abscess of the appendix vermiformis and from iliac abscess I think psoitis may be distinguished by the peculiar position of the limb in the latter, viz: flexed upon the abdomen and rotated outwards.)

*Prognosis.*—Psoitis is a very grave disease. Dr. Kyll thinks the prognosis very favorable, all his five patients having recovered, and yet he admits that he is not sure whether the seat of the disease was in the psoas muscle, the lumbar vertebræ or the peloric cellular tissue. Psoitis does sometimes terminate in recovery, as is shown by a case reported by La Motte. Fluctuation was felt deeply along the vertebræ and loins between the last rib and ilium. A large incision was made and six pounds of pus extracted. After five months the patient recovered. There was some doubt, however, as to the nature of the disease. (Prof. Willard Parker informs me that the two cases which have come under his observation recovered. The case reported by Dr. C. W. Parsons also recovered.)

In the collections of pus which form in the pelvic cavity, the bursting of the abscess into the intestines is considered favorable, but in psoitis such an event is considered unfavorable, as is shown by the two cases already mentioned.

*Treatment.*—As soon as the patient complains of pain along the track of the psoas muscle and other circumstances enable us to predict inflammation, general bleeding is recommended, repeated applications of leeches and cups to the loins and groins, fomentations, frictions with mercury, ammonia, iodine, etc. Finally, blisters and caustics may be employed. If suppuration takes place the painful parts should be covered with warm poultices. Constipation should be relieved by a purgative or laxative. If the abscess points at the surface it should be opened.

